# The Tryptophan System in Cocaine-Induced Depression

**DOI:** 10.3390/jcm9124103

**Published:** 2020-12-19

**Authors:** Francina Fonseca, Joan-Ignasi Mestre-Pintó, Àlex Gómez-Gómez, Diana Martinez-Sanvisens, Rocío Rodríguez-Minguela, Esther Papaseit, Clara Pérez-Mañá, Klaus Langohr, Olga Valverde, Óscar J. Pozo, Magí Farré, Marta Torrens, on behalf of NEURODEP GROUP

**Affiliations:** 1 Addiction Research Group (GRAd), Neuroscience Research Program, Hospital del Mar Medical Research Institute (IMIM), 08003 Barcelona, Spain; MFFonseca@parcdesalutmar.cat (F.F.); roromin1@hotmail.com (R.R.-M.); 2 Institut de Neuropsiquiatria i Addiccions, Hospital del Mar, 08003 Barcelona, Spain; 98459@parcdesalutmar.cat; 3 Department of Psychiatry and Department of Pharmacology, School of Medicine, Universitat Autònoma de Barcelona (UAB), 08290 Cerdanyola del Vallès, Spain; epapaseit.germanstrias@gencat.cat (E.P.); cperezm.mn.ics@gencat.cat (C.P.-M.); mfarre.germanstrias@gencat.cat (M.F.); 4 Department of Experimental and Health Sciences (CEXS), Universitat Pompeu Fabra, 08002 Barcelona, Spain; agomez@imim.es; 5 Integrative Pharmacology and Systems Neuroscience Research Group, Neuroscience Research Programme, Hospital del Mar Medical Research Institute (IMIM), 08003 Barcelona, Spain; klangohr@imim.es (K.L.); opozo@imim.es (Ó.J.P.); 6 Clinical Pharmacology Department, Hospital Universitari Germans Trias i Pujol (IGTP), 08003 Badalona, Spain; 7 Department of Statistics and Operations Research, Universitat Politècnica de Barcelona Barcelonatech, 08034 Barcelona, Spain; 8 Neurobiology of Behaviour Research Group (GReNeC-NeuroBio), Department of Experimental and Health Sciences, Universitat Pompeu Fabra, 08002 Barcelona, Spain; olga.valverde@upf.edu; 9 Neurobiology of Behaviour Research Group, Neuroscience Research Programme, Hospital del Mar Research Institute (IMIM), 08003 Barcelona, Spain

**Keywords:** primary/substance-induced depression, cocaine use disorder, tryptophan, serotonin, kynurenine

## Abstract

Major depression disorder (MDD) is the most prevalent psychiatric comorbid condition in cocaine use disorder (CUD). The comorbid MDD might be primary-MDD (CUD-primary-MDD) or cocaine-induced MDD (CUD-induced-MDD), and their accurate diagnoses and treatment is a challenge for improving prognoses. This study aimed to assess the tryptophan/serotonin (Trp/5-HT) system with the acute tryptophan depletion test (ATD), and the kynurenine pathway in subjects with CUD-primary-MDD, CUD-induced-MDD, MDD and healthy controls. The ATD was performed with a randomized, double-blind, crossover, and placebo-controlled design. Markers of enzymatic activity of indoleamine 2,3-dioxygenase/tryptophan 2,3-dioxygenase, kynurenine aminotransferase (KAT) and kynureninase were also established. Following ATD, we observed a decrease in Trp levels in all groups. Comparison between CUD-induced-MDD and MDD revealed significant differences in 5-HT plasma concentrations (512 + 332 ng/mL vs. 107 + 127 ng/mL, *p* = 0.039) and the Kyn/5-HT ratio (11 + 15 vs. 112 + 136; *p* = 0.012), whereas there were no differences between CUD-primary-MDD and MDD. Effect size coefficients show a gradient for all targeted markers (*d* range 0.72–1.67). Results suggest different pathogenesis for CUD-induced-MDD, with lower participation of the tryptophan system, probably more related to other neurotransmitter pathways and accordingly suggesting the need for a different pharmacological treatment approach.

## 1. Introduction

Major depression disorder (MDD) is the most prevalent psychiatric comorbid condition in subjects with cocaine use disorder (CUD), and its treatment constitutes a challenge [1]. In clinical practice, the co-occurrence of substance use hinders both the diagnosis of depressive symptoms and the differentiation between primary depressive and cocaine-induced episodes. Indeed, such a distinction could prove crucial in improving diagnostics and treatment, and therefore the prognoses [2,3]. Accordingly, there is a growing interest in putative biomarker research that enhances a better approach to this comorbid pathology.

**Figure 1:**
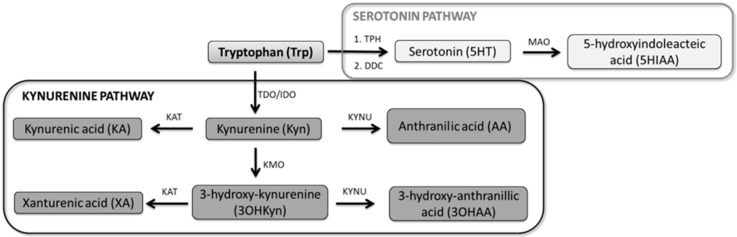
Tryptophan metabolic pathways. Metabolites involved in the tryptophan system including serotonin and kynurenine pathways. TPH, tryptophan hydroxylase; DDC, aromatic L-aminoacid decarboxylase; MAO, monoamine oxidase; IDO, indoleamine 2,3-dioxygenase; TDO, tryptophan 2,3-dioxygenase; KAT, kynurenine aminotransferase; KYNU, L-kynurenine hydrolase; and KMO, kynurenine 3-monooxygenase.

Depression and substance use disorder (SUD) share some common neurobiological pathways [4] such as the monoaminergic, endocannabinoid and, inflammatory ones. Traditionally, the serotoninergic (5-HT) pathway has been the most studied in major depression, and there is clear evidence of its implication in the neurobiology of primary-MDD [5]. There are various models to investigate 5-HT pathways, including modulation of the tryptophan (Trp) system, which has been reported as playing a critical role in both the pathogenesis of depression [6] and SUD [5]. Trp undergoes two main metabolic pathways (Figure 1). On the one hand, by the subsequent action of tryptophan hydroxylase and aromatic L-aminoacid decarboxylase, Trp is converted into 5-HT. Reduced 5-HT synthesis and bioavailability due to the depletion of Trp (5-HT precursor) has been postulated as a tool to study MDD mechanisms and potential biomarkers. The acute tryptophan depletion test (ATD) is a standardized method of reducing brain 5-HT through the administration of large neutral amino acids (LNAAs). This limits the transport of endogenous Trp across the blood–brain barrier by competition with other LNAAs and subsequently decreases serotonergic neurotransmission. It has shown to be useful in studying the effect of 5-HT on mood [7,8,9]. After a depletion of Trp, and the consequent temporary reduction in 5-HT levels, a lowering in mood has been observed in patients with previous MDD history, but not in those without a personal or familial diagnosis of depression [10]. On the other hand, through the kynurenine pathway, Trp is transformed into kynurenine (Kyn) by the action of indoleamine 2,3-dioxygenase (IDO) and tryptophan 2,3-dioxygenase (TDO). The dysregulation of metabolites in both pathways has been associated with MDD. In this regard, a decrease in the synthesis of 5-HT has been linked to depressive symptoms, and a stimulation of IDO followed by an increase in Kyns can trigger depression [11,12,13]. Raised IDO activity has been associated with MDD [11,14,15,16]. Kyn has also been involved in several neuropsychiatric disorders, including depression and schizophrenia-like cognitive deficits (for a revision see Savitz et al., 2020) [17]. Many Kyns are neuroactive, modulating neuroplasticity and/or exerting neurotoxic effects in part through their effects on N-methyl-D-aspartate (NMDA) receptor signaling and glutamatergic neurotransmission. Their involvement in neuropsychiatric disorders is related to inflammation; this system has been described as being a regulator of the immune system. In addition, depression has been associated with inflammatory factors [18,19], for instance, increased levels of pro-inflammatory cytokines including interleukin (IL)-6, tumor necrosis factor (TNF) α, and IL-1β. Moreover, elevated concentrations of acute phase proteins have been reported in patients with MDD [20] and there are studies describing the response to immunotherapy in depression [21]. On the other hand, the Kyn system and SUD have not been researched as extensively as depression, although it has been observed that MDD could be involved in SUD mediated by the glutamatergic system. Kyn acid is an antagonist of the NMDA receptor, therefore increasing its concentrations has been proposed as a possible treatment strategy for the craving and relapse in alcohol addiction in animal models [22]. Although some authors have reported no significant differences in Kyn levels between MDD and healthy controls [23], it is generally agreed that higher Kyn levels are found in MDD subjects [24,25,26]. Methodological variations, as the type of sample or statistical analyses, could explain the differences found in the studies and reviews. The majority of studies are based on analyses that look for statistically significant differences in concentrations and ratios rather than effect sizes. Remarkably, Kyn levels have been observed to correlate with the addition of celecoxib, a cyclooxygenase-2 inhibitor, nonsteroidal anti-inflammatory drug indicated for the treatment of osteoarthritis and rheumatoid arthritis [27,28] in managing MDD [29]. They have also been described as predictors of acute responses to ketamine treatment for severe depression [30]. Despite the increasing evidence regarding the role of Kyn in MDD, no findings with respect to CUD and CUD-induced-MDD have been reported.

Furthermore, the 5-HT hypothesis in depression has been empirically confirmed through the use of selective serotonin reuptake inhibitors (SSRI), antidepressants that block the presynaptic 5-HT transporter and thus increase 5-HT concentrations in the synapses. When considering substance-induced MDD, however, this theoretical basis is less clear due to the lack of response to SSRI in comorbid MDD [31], suggesting the involvement of other neurotransmitter systems in the neurobiology of induced depression. Comorbid depression is one of the most relevant indicators of a poor prognosis in CUD patients, therefore providing effective treatment is crucial.

We have hypothesized that differences on markers of tryptophan pathways between cocaine-induced depression (CUD-induced-MDD), CUD-primary depression (CUD-primary-MDD) and MDD, are significant and of medium–large magnitude. To test this hypothesis, we have designed a study aimed at assessing the Trp system in both the 5-HT pathway, through ATD, and the Kyn pathway, in subjects diagnosed CUD-primary-MDD, CUD-induced-MDD, MDD, and matched healthy controls (HC).

## 2. Experimental Section

### 2.1. Subjects

A total of 35 subjects participated in the study. All diagnoses were performed according to DSM-IV-TR criteria [32]. Patients were recruited at the addiction treatment facilities of the Institute of Neuropsychiatry and Addiction at Parc Salut Mar in Barcelona (Spain). 

HC were included from a database of healthy subjects willing to participate in medical research projects at the Pharmacology Unit of the IMIM-Hospital del Mar Medical Research Institute, Barcelona (Spain).

Inclusion criteria were: both genders, age > 18 years, Caucasian origin, and body mass index 19–29 kg/m^2^. In the MDD groups (primary and induced), the most recent episode had to be in remission, and 17-item Hamilton Depression Rating Scale (HDRS) [33] score ≤ 6. In the CUD groups, subjects had to have maintained at least 4 weeks of substance abstinence prior to the trial as confirmed by detection in random urine controls. 

Exclusion criteria included: cognitive or language limitations that precluded evaluations (based on the clinical criteria of the evaluators), pregnant or breastfeeding women, use of anti-inflammatory drugs or monoamine oxidase inhibitors (MAOIs), and any medical problem that could interfere in the study procedures. In the comorbid CUD and MDD groups: any psychiatric disorder in Axis I other than MDD, and/or any substance use disorders other than cocaine or nicotine. In the HC group: any psychiatric disorder in Axis I, family history of depressive disorder, and any substance use disorder (except nicotine).

The clinical protocol was approved by the local Research Ethical Committee CEIC-Parc de Salut Mar, Barcelona, Spain (2009/3494/I and 2012/4751/I) and the study was conducted in accordance with the Declaration of Helsinki and Spanish laws concerning clinical research. Volunteers were financially compensated. All subjects gave written informed consent prior to their participation in the study. 

### 2.2. Clinical Assessments

A close-ended questionnaire was used to record patients’ sociodemographic characteristics, family history, medical assessment, history of substance use, and previous psychiatric treatment. Depression severity was evaluated with the 17-item Spanish version of the HDRS [34].

### 2.3. Psychiatric Assessment 

SUD and non-SUD were diagnosed according to DSM-IV-TR criteria [32] using the Spanish version of the Psychiatric Research Interview for Substance and Mental Disorders IV (PRISM-IV) [35]. The PRISM is a semi-structured interview designed to differentiate primary disorders, SUD, and the expected effects of intoxication and withdrawal when evaluating current and life-time DSM-IV-TR disorders. Diagnoses obtained through the PRISM interview have shown good to excellent validity and test–retest reliability for primary-MDD and substance-induced MDD [35,36].

### 2.4. Acute Tryptophan Depletion Test (ATD)

In one of the sessions, subjects were given an amino acid mixture lacking Trp. ATD-session: L-alanine 5.5 g, L-arginine 4.9 g, L-cysteine 2.7 g, glycine 3.2 g, L-histidine 3.2 g, L-isoleucine 8.0 g, L-leucine 13.5 g, L-lysine 11 g, L-methionine 3.0 g, L-proline 12.2 g, L-phenylalanine 5.7 g, L-serine 6.9 g, L-threonine 6.9 g, L-tyrosine 6.9 g, and L-valine 8.9 g. Non-ATD: L-Trp: 2.3 g was administered. The order of ATD and non-ATD sessions was randomly determined, and investigators and subjects were blinded to the amino acid content of the mixture. 

Subjects were admitted to the IMIM Clinical Research Unit facilities at 08.00 after an overnight fast (from 21.00 h). The day before the experimental sessions they were required to follow a low-Trp diet [7,9] (see Supplementary Material). Subjects presenting nicotine addiction were treated during the experimental session with patches according to their nicotine daily dose. A urine sample was collected for drug testing (Instant-View^®^, Multipanel 10 Test Drug Screen, Alfa Scientific Designs Inc., Poway, CA, USA). Participants were required to be drug-free before inclusion in each experimental session. The capsules and amino acid drink with/without tryptophan were administered between 08:15 and 08:30. The subjects remained sitting/lying in a calm laboratory environment during the session, with restricted social interactions. Blood for platelet-poor plasma was obtained at different time points: basal and 5 h. It was collected by venipuncture in a 4 mL plastic tube containing EDTA and immediately centrifuged at 12,000× *g* for 10 min. The remaining platelet-poor plasma was divided into aliquots and stored at −20 °C until analysis. 

After 5 h of the mixture intake, all subjects were given an enriched Trp diet (containing pasta, chicken, and banana). To control for undesired Trp depletion side effects (such as sustained depressed mood or suicidal ideation) patients remained on the laboratory premises until 17:00 and were requested to return 24 h after session commencement. 

### 2.5. Selection of the Serotonin–Kynurenine Pathway Biomarkers

Markers belonging to the serotonin–kynurenine pathway were quantified by a previously validated method based on liquid chromatography tandem mass spectrometry (LC-MS/MS) [37]. Six markers of the serotonergic pathway were included in the present study. Plasmatic levels of 5-HT and 5-hydroxyindoleacteix acid (5HIAA) were measured and some amino acid ratios were calculated. Values of 5-HT/Trp and 5HIAA/5-HT were used to assess the enzymatic activity of tryptophan hydroxylase (TPH) and MAO, respectively (Figure 1). Kyn/5-HT provided information about the preferential pathway in Trp metabolism and 5HIAA/Trp was calculated as an indicator of the whole serotoninergic pathway. Additionally, seven markers belonging to the kynurenine pathway were evaluated. The plasmatic concentrations of Kyn, kynurenic acid (KA), and anthranilic acid (AA) were measured. The enzymatic activity of IDO/TDO, kynurenine aminotransferase) KAT, and kynureninase was established by the calculation of Kyn/Trp, KA/Kyn, and AA/Kyn ratios, respectively (Figure 1).

### 2.6. Statistical Analysis

A descriptive analysis of all variables of interest was carried out separately in each of the four study groups. For this purpose, the mean, median, standard deviation, and range were calculated. Repeated measures ANOVA models were used to analyze the changes after 5 h of both the tryptophan and the Hamilton Depression Rating Scale scores. The models included ATD and group condition as main factors as well as all two-way and three-way interactions and the computation of simultaneous confidence intervals and adjusted *p*-values to guarantee that a family-wise error rate of 0.05 was based on the multivariate t distribution of the vector of test statistics. 

Concerning the targeted markers, given the skewed distribution of most them, these data were log-transformed prior to the inferential analyses. Next, 1-way ANOVA models were fitted to compare the study groups with respect to the mean of the log-transformed variables. The model assumptions (homoscedasticity and normally distributed residuals) were checked with residual plots as well as with the Levene test (homoscedasticity) and the Kolmogorov–Smirnov test, respectively. If assumptions held and group differences were statistically significant, the Tukey test was applied for the post-hoc pairwise comparisons. Otherwise, nonparametric analyses were carried out. For this purpose, the Kruskal–Wallis test was used to compare the study groups with respect to the median, and the post-hoc comparisons (if applied) were performed with Dunn’s Test [38]. Cohen’s *d* was used to quantify the effect size of the pairwise differences among study groups (small: *d* ≤ 0.20; medium: *d* ≥ 0.50; large: *d* ≥ 0.80; very large: *d* ≥ 1.30). Cohen’s *d* is a standardized score, analogous to a z score. Following Cohen’s effect-size conventions, only differences higher than a medium effect size (*d* ≥ 0.50) were considered of relevance [39,40].

All data were analyzed using the statistical software R, version 3.4.3. (Vienna, Austria; http://www.rproject.org). In the case of the group comparisons, statistical significance was set at 0.05 (to protect against Type-I errors), and for model assumption tests at 0.1 (to protect against Type-II errors). 

## 3. Results

### 3.1. Demographic and Clinical Characteristics 

A total of 35 subjects were included in the study. The main sociodemographic and clinical characteristics of the sample are described in Table 1. The final groups were: 8 HC, 8 CUD-Induced-MDD, 14 CUD-Primary-MDD, and 5 MDD.

The groups diagnosed with primary depression had a similar age at the onset of depressive disorders, and the incidence of previous depressive episodes did not differ substantially among all groups. The CUD-primary-MDD group showed a highly prevalent history of family depression. The number of subjects under antidepressant treatment was lower in the CUD-induced-MDD group (37.5%) than the CUD-primary-MDD group (57.1%). 

Regarding substance use data, there were no differences in the age of cocaine use onset. More than 70% of the sample of the CUD groups presented a nicotine use disorder, whereas the MDD and HC groups were non-smokers (Table 1).

### 3.2. Acute Tryptophan Depletion Test (ATD)

**Figure 2:**
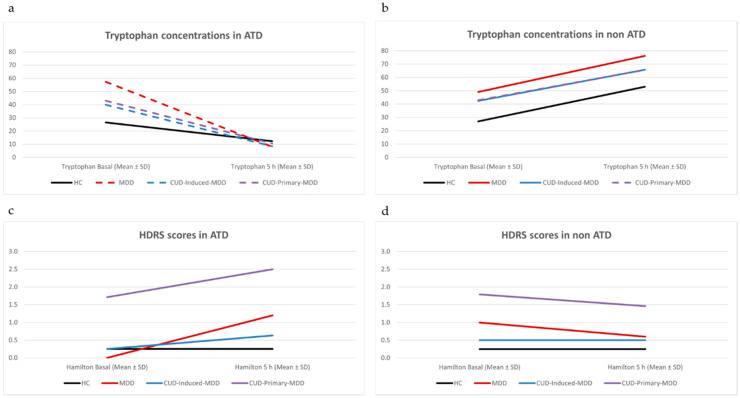
Variations in tryptophan concentrations and Hamilton Depression Rating Scale (HDRS) scores in both acute tryptophan depletion (ATD) and non-ATD tests. Dotted lines indicate significant changes (according to repeated measures ANOVA) between basal and 5 h follow up. 2a *p* < 0.020, 2b *p* = 0.030. (**a**) Tryptophan concentrations in ATD. (**b**) Tryptophan concentrations in non ATD. (**c**) HDRS scores in ATD. (**d**) HDRS scores in non ATD.

Based on the repeated measures ANOVA model, except for the HC, the levels of Trp in all the ATD groups were significantly decreased in a similar way (Figure 2a). In the non-ATD test, there was a significant increase in Trp levels only in CUD-Primary-MDD between baseline and 5 h (Figure 2b). In the non-ATD session there was only a significant increase in Trp levels in CUD-primary-MDD group between baseline and 5 h (Figure 2d). Regarding HDRS scores, there were no significant changes between basal and 5 h in either session (Figure 2c,d). See Table S1 of Supplementary Material.

### 3.3. Kynurenine Pathway

As a first step, the basal values obtained for the 13 targeted markers (serotonin and Kyn pathways) in the HC group were compared with those in the MDD to select the most appropriate markers for MDD. Results for the values for each of the selected markers are summarized in Table 2.

#### 3.3.1. Controls vs. MDD

**Figure 3:**
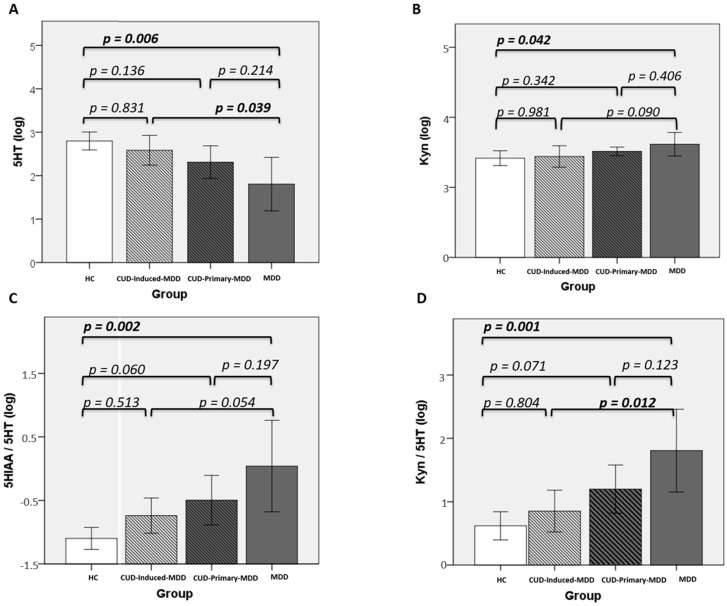
Variations observed in the four selected markers for the CUD-induced/primary depression groups compared with HC and MDD. (**A**) 5-HT, (**B**) Kyn, (**C**) 5HIAA/5-HT, and (**D**) Kyn/5-HT. Post-hoc comparisons were performed with Dunn’s Test.

The main differences were observed in the plasmatic concentration of the two principal tryptophan metabolites, 5-HT and Kyn: the former was significantly decreased in MDD (*p* = 0.006) whilst the latter was found to be increased (*p* = 0.042) (Figure 3A,B). Differences were observed in 5HIAA/5-HT (marker of MAO enzymatic activity) and Kyn/5-HT (marker of the balance between both pathways) (Figure 3C,D). Both ratios were significantly raised (*p* = 0.002 and *p* = 0.001, respectively) for the MDD volunteers. No significant differences were reported for the rest of the tryptophan metabolites in either the 5-HT or Kyn pathways. Based on these results, 5HIAA/5-HT and Kyn/5-HT were selected as potential depression markers. The effect size coefficients of all these comparisons were large (*d* ≥ 1.24) (Table 3).

#### 3.3.2. Cocaine Use Disorder Groups

CUD-induced-MDD showed a significantly higher concentration of 5-HT (*p* = 0.039) when compared with MDD levels, and a lower, but not significant, Kyn concentration was found (*p* = 0.090) (Figure 3A,B). The values of the ratios presented significant differences for Kyn/5-HT (increased in MDD, *p* = 0.012) but only a trend for 5HIAA/5-HT (*p* = 0.054) (Figure 3C,D). Remarkably, no significant differences were observed in any marker for the comparison between HC and CUD-induced-MDD.

CUD-induced-MDD showed large or very large differences (*d* > 1) when comparing all targeted markers with MDD (Table 3) in terms of magnitude of the effect. Additionally, CUD-primary-MDD presented medium to large differences from MDD (*d* ≥ 0.72 ≤ 0.90) when performing the same comparisons.

#### 3.3.3. Overall Perspective

When looking at the results as a whole, with MDD group as reference, effect size coefficient shows a gradient where for all targeted markers the biggest magnitude of the effect is HC (*d* ranging from 1.24 to 1.67), followed by CUD-Induced-MDD (*d* ranging from 1.04 to 1.47), and ending with CUD-Primary-MDD (*d* ranging from 0.72 to 0.90).

Taking HC group as reference, the same gradients appear where largest magnitude of the effect is MDD (*d* ranging from 1.24 to 1.67), followed by CUD-primary-MDD (*d* ranging from 0.73 to 0.91), and ending with CUD-induced-MDD (*d* ranging from 0.25 to 0.83), except for 5-HT where CUD groups flip their positions (CUD-induced-MDD d= 0.54 and CUD-primary-MDD *d* = 0.38).

## 4. Discussion

The present study suggests a preservation of the Trp system in CUD-induced-MDD because its results are similar to the HC and significantly different from MDD in 5-HT and Kyn/5-HT. Furthermore, the results also show the already known differences between HC and MDD in 5-HT and Kyn values (and ratios). All this points to the existence of similar patterns in MDD and CUD-primary-MDD groups on the one hand, and between HC and CUD-induced-MDD groups on the other.

To the best of our knowledge, this is the first study to evaluate ATD in the CUD-primary/induced-MDD. In 1995, Satel et al. studied the implications of a reduction in Trp levels in the addiction symptoms of CUD patients, mainly referring to drug craving. They observed that following depletion there was a decrease in craving scores [41]. Cocaine craving, and the subsequent loss of control, is a key factor associated with relapse, and research has been carried out regarding the neurobiological mechanisms involved [42]. With respect to comorbid MDD, one study assessed the effect of ATD on depression scores depending on smoker status [43]. The authors proposed that, apart from 5-HT, other neurotransmitters could be involved, probably the acetylcholine pathway and receptors; however, they did not discriminate between primary and substance-induced MDD. Findings from the functional test follow similar trends when studying the biological markers.

Our results pose that subjects with CUD-induced-MDD present differences at the biochemical level when compared with MDD. Gradients showed by the effect size coefficients for all targeted markers suggest that biochemical alterations in both the 5-HT and Kyn pathways in CUD-primary-MDD resemble those observed in MDD. Moreover, the changes suffered in these pathways by patients with CUD-induced-MDD are less prominent and closer to HC values.

According to these results and previously published research [44] it can be presumed that the biochemical basis of induced-MDD differs from primary-MDD. The neurobiological basis for induced depression in CUD does not seem to be primarily mediated by 5-HT dysfunction and other neurotransmitter pathways may be involved. In this regard, research has presented promising results showing alterations in dopamine pathways [45], the cocaine and amphetamine-regulated transcript (CART) peptide [46], and neurotrophins [47]. Cocaine reinforcing effects are directly related to the dopamine concentration in the mesocortical system. As a result, the pathogenesis of CUD-induced-MDD could be associated with changes in the dopaminergic system, also associated with motivation and anhedonia [48] and with changes in Brain-derived Neurotrophic Factor (BDNF) signaling [47,49].

There are several limitations to this study. Firstly, the sample size was small for all groups; the strict inclusion criteria made it difficult to find pure cocaine/depressed only patients. For this reason, although our results suggest biochemical differences between MDD and CUD-induced-MDD, such findings should be confirmed by the analysis of a larger set of samples. A second limitation is that the MDD patients were under remission. Although this helped in the depletion test, it could have contributed to the moderate differences observed in some comparisons. It is expected that greater differences could be obtained in the analysis of some of these markers in patients suffering from moderate–severe depressive symptoms. Additionally, we cannot discard the fact that significant differences can be found for some additional biomarkers when analyzing samples from patients with depressive symptoms. Such could be the case of the ratio Kyn/Trp (targeting TDO and IDO activities) that has been reported to be increased in SUD patients with depressive symptoms [50], although no significant differences were found in our study. An added limitation which should be resolved in further investigation is the lack of an experimental group made up of subjects diagnosed with CUD without major depression disorder. Finally, the small sample size also hampered a proper evaluation of the effect of gender on the results. Although performing such studies based on the tryptophan depletion test would be difficult, the basal bioanalytical changes would allow detailed assessment of several aspects including gender effect. Depressive disorders are more common in women than men, moreover, depression associated with addictive disorders (primary or induced) is more prevalent in women with SUD than men, and more frequent than expected in women without any SUD [51]. Differences have also been found in clinical presentation and some neurobiological markers [52] including the Kyn pathway. For example, in a study in a Finnish population, IDO levels associated with depressive symptoms differed between the genders [15]. In this regard, we tried to minimize the impact of gender differences by performing the tests in all the women during their follicular phase.

Despite such limitations, results of this study indicate a different pathogenesis for CUD-induced depression. The participation of the Trp system varies and is probably more related to other neurotransmitter pathways. The lack of efficacy of SSRI in the treatment of dual depression, at least in CUD patients, and the efficacy of tricyclic antidepressants, is probably due to a greater range of neurotransmitters system effects through their mechanism of action [3,31]. Further studies are needed to confirm the role of the Kyn pathway and to explore other neurobiological systems in CUD patients with comorbid depression to improve treatment approach and prognosis.

The results of this study are a first step to the better knowledge of the differential mechanisms in primary and induced MDD. As previously mentioned, depression and substance use disorders represent 7% of the disease burden measured in disability-adjusted life years worldwide, they also contribute to general mortality with no contemporary decrease [53]. The joint presence of an MDD and SUD increases the severity of both disorders due to their high prevalence and poor prognosis [3,54]. Although induced MDD has been traditionally appraised as a minor condition that could improve spontaneously with substance abstinence, research has demonstrated that it could imply a worse prognosis for both affective and SUD [55]. Samet et al. (2013) [55] reported that patients with substance induced MDD showed a higher risk of relapse than those with primary-MDD. Moreover, longitudinal studies have reported that primary-MDD has been detected in patients initially diagnosed with induced-MDD [56]. Finally, there are treatment implications that could benefit from a thorough diagnosis of primary and induced-MDD as differential responses to antidepressants in both type of depression [57] have been described, with poor response to SSRI in the latter. Our results concur; CUD-primary-MDD showed a similar pattern of Trp system to MDD, and CUD-induced-MDD 5-HT and Kyn/5-HT were significantly different from MDD groups. Our hypothesis is that tryptophan metabolic pathways could be less involved in the pathogenesis of induced depression. Additionally, the kynurenine pathway can be useful as a biomarker in patients with any kind of depression; nevertheless, other neurobiological systems (dopamine, glutamate, endogenous opioid, and endocannabinoid systems) should be explored to define the pathophysiology of induced depressions.

## 5. Conclusions

The tryptophan system, including the serotonin and kynurenine pathways, might help in differentiating among primary-depressive episodes and those which are cocaine-induced in subjects with CUD. This differentiation could be crucial in improving treatment approach and prognosis.

## Figures and Tables

**Table 1:** Sociodemographic and clinical characteristics of the sample at baseline (*n* = 35).

	HC	CUD-Induced-MDD	CUD-Primary-MDD	MDD
	*n* = 8	*n* = 8	*n* = 14	*n* = 5
Sex (Male *n*)	5 (50)	6 (75)	12 (85.7)	5 (100)
Age (Mean ± SD)	32.13 ± 3.94	38 ± 12.17	44.21 ± 8.26	43.80 ± 13.9
Civil status (% Single)	62.5	62.5	35.7	40
Work status (% Employed)	25	37.5	50	40
Depression (MDD)				
Age of onset first primary-MDD (Mean ± SD)	NA	NA	34.29 ± 10.56	36.4 ± 11.97
Age of onset first induced-MDD (Mean ± SD)	NA	34.01 ± 12.47	NA	NA
Number of episodes (Mean ± SD)	NA	6.63 ± 8.23	2.08 ± 1.04	1.6 ± 0.89
Months since last episode (Mean ± SD)	NA	22.75 ± 31.23	36.29 ± 55	34.4 ± 40.9
Family history of depression (%)	NA	37.5	71.4	80
Current antidepressant treatment (%)	NA	37.5	57.1	100
Age of onset CUD	NA	26.75 ± 10.29	31.43 ± 7.39	NA
Age of cocaine problematic use	NA	26.38 ± 9.61	31.21 ± 7.43	NA
Maximum cocaine abstinence period (Months)	NA	19.50 ± 18.42	37.77 ± 54.17	NA
No. 1st and 2nd degree relatives with CUD	0	0	0.64 ± 0.93	0
Nicotine use disorder (%)	0	75	78.6	0

HC, healthy controls; MDD, major depression disorder; CUD, cocaine use disorder; NA, not applicable.

**Table 2:** Basal values for the serotonin–kynurenine pathways (ng/mL for concentrations and adimensional for ratio values).

	HC	CUD-Induced-MDD	CUD-Primary-MDD	MDD
	*n* = 8	*n* = 8	*n* = 14	*n* = 5
Kyn	2716 ± 847	2971 ± 1171	3358 ± 894	4293 ± 1424
KA	247 ± 95	272 ± 135	304 ± 128	370 ± 205
AA	64 ± 23	79 ± 61	54 ± 32	32 ± 12
5-HT	729 ± 456	512 ± 332	521 ± 584	107 ± 127
5-HIAA	52 ± 14	87 ± 76	68 ± 21	75 ± 15
Kyn/Trp	0.016 ± 0.004	0.019 ± 0.006	0.021 ± 0.006	0.027 ± 0.015
KA/Kyn	0.093 ± 0.028	0.091 ± 0.026	0.094 ± 0.037	0.084 ± 0.021
AA/Kyn	0.026 ± 0.014	0.031 ± 0.023	0.016 ± 0.009	0.008 ± 0.004
KA/AA	4.2 ± 1.6	6.8 ± 7.3	9.4 ± 10.2	13.8 ± 9.4
Kyn/5-HIAA	56 ± 22	43 ± 17	58 ± 37	60 ± 16
5-HT/Trp	0.0042 ± 0.0022	0.0032 ± 0.0018	0.0031 ± 0.0033	0.0008 ± 0.0003
5-HIAA /5-HT	0.088 ± 0.038	0.26 ± 0.29	0.91 ± 1.12	2.08 ± 2.67
5-HT/Kyn	0.286 ± 0.188	0.183 ± 0.114	0.164 ± 0.184	0.027 ± 0.032
5-HIAA/Trp	0.00031 ± 0.00009	0.00053 ± 0.00038	0.00044 ± 0.00015	0.00043 ± 0.00014
Kyn/5-HT	4.8 ± 2.5	11 ± 15	44 ± 55	112 ± 136

Note: Descriptive data are presented as mean ± standard deviation (SD). HC, healthy controls; MDD, major depression disorder; CUD, cocaine use disorder; Kyn, kynurenine; KA, kynurenic acid; AA, anthranilic acid; 5-HT, serotonin; 5-HIAA, 5-hydroxyindoleacetic acid; Trp, tryptophan.

**Table 3:** Effect size coefficient (Cohen’s *d*) comparisons between groups in the four targeted markers.

		CUD-Induced-MDD (*n* = 8)	CUD-Primary-MDD (*n* = 14)	MDD (*n* = 5)
5-HT	HC (*n* = 8)	0.54	0.38	1.67 **
	CUD-Induced-MDD	—	0.02	1.47 *
	CUD-Primary-MDD	—	—	0.80
Kyn	HC	0.25	0.73	1.44 *
	CUD-Induced-MDD	—	0.39	1.04
	CUD-Primary-MDD	—	—	0.90
5-HIAA/5-HT	HC	0.83	0.91	1.24 **
	CUD-Induced-MDD	—	0.71	1.12
	CUD-Primary-MDD	—	—	0.72
Kyn/5-HT	HC	0.58	0.88	1.31 **
	CUD-Induced-MDD	—	0.73	1.22 **
	CUD-Primary-MDD	—	—	0.83

Cohen’s effect size: small (*d* > 0.20), medium (*d* > 0.50), large (*d* > 0.80), and very large (*d* > 1.30). HC, healthy controls; MDD, major depression disorder; CUD, cocaine use disorder; Kyn, Kynurenine; 5-HT, Serotonin; 5-HIAA, 5-hydroxyindoleacetic acid. * *p* < 0.05 ** *p* < 0.01.

## Supplementary Materials

The following are available online at https://www.mdpi.com/2077-0383/9/12/4103/s1, Table S1. ADT tryptophan and Hamilton Depression Rating Scale scores, Appendix 1. NEURO-DEP Study diet.
