# Supplementary material for: The Tryptophan System in Cocaine-Induced Depression
Source: J Clin Med. 2020 Dec 19;9(12):4103. doi: 10.3390/jcm9124103 (PMC7766966; doi:10.3390/jcm9124103)
Supplement: Supplementary file 1 [file jcm-09-04103-s001.pdf]

Supplementary material

Table 1. S. ADT tryptophan and Hamilton Depression Rating Scale scores.

	HC	MDD	CUD-Induced-MDD	CUD-Primary-MDD
**ADT session**	***N* = 8**	***N* = 5**	***N* = 8**	***N* = 14**
Tryptophan Basal	26.54±25.21	57.32±9.17	39.98±22.17	41.71±20.86
Tryptophan 5h[Ψ]	12.24±6.64	8.02±5.14	8.49±3.71	10.01±5.79
Effect Size	0.78	6.63	1.98	2.07
Hamilton Basal	0.25±0.71	0	0.25±0.71	1.71±1.82
Hamilton 5h*	0.25±0.71	1.20±1.79	0.63±0.92	2.5±1.83
Effect Size	0.00	0.95	0.46	0.43
**Non-ADT session**				
Tryptophan Basal	27.03±24.19	49.06±11.18	42.35±20.49	42.86±22.23
Tryptophan 5h[ΨΨ]	53.01±60.17	76.20±17.50	65.75±38.45	65.66±39.23
Effect Size	-0.57	-1.85	-0.76	-0.72
Hamilton Basal	0.25±0.71	1.00±2.24	0.50±0.93	1.79±1.81
Hamilton 5h	0.25±0.71	0.60±1.34	0.50±0.76	1.46±1.27
Effect Size	0.00	-0.22	0.00	-0.21

Note: Descriptive data are presented as mean ± standard deviation (SD)

ADT: acute depletion test; HC: Healthy controls; MDD: major depression disorder; CUD: cocaine use disorder

[Ψ]According to repeated measures ANOVA, differences between basal and 5h $p < 0.020$, except HC ($p = 0.776$) in ADT.

[ΨΨ]According to repeated measures ANOVA, differences between basal and 5h $p = 0.030$ in CUD-Primary-MDD in non-ADT.

Appendix 1. NEURO-DEP Study diet

As we have previously informed you, participants in the NEURO-DEP Study must meet two requirements to participate in the two experimental sessions of the study:
1. Follow a special diet the day before the experimental sessions.
2. Attend the mornings of the experimental sessions in a fasting state.

Please read carefully the allowed and prohibited foods during those 24 hours prior to each session. Choose the right foods in your case and consider the amounts allowed.

Allowed foods: oil, sugar, any vegetable (100g/2 times a day) (except watercress, pumpkin, and spinach) and fruit (apple, orange, watermelon, and melon are recommended). Rice and pasta in small quantities (maximum 30g/ration in a day), infusions. Fresh cheese (the amount you want), pears (the amount you want).

Forbidden foods: eggs, dairy products - milk, cheese and yoghurt (only milk in quantity less than 100ml/day, fresh cheese is allowed), meat, fish, soya (plant, flour, and milk), legumes, wholegrain cereals (pasta and rice), nuts and raisins. Bananas, strawberries, avocados, chocolate and cocoa. BREAD. Alcohol (beer, wine, spirits), coffee and tea

Example Diet

Breakfast:
Full-fat milk (100ml milk = half a glass) + 10g of sugar
Two pieces of fruit or five cookies (wholegrain type prohibited)
Attention! Do not exceed the recommended amount of milk.
Lunch
1º Choose between
- Vegetables (100g) such as green beans, potatoes, white asparagus with oil, vinegar, garlic or spices to taste. No sauces.
- Mashed potato (80g of cooked potato)

2º Rice/Pasta (30g) with tomato (30g).
Dessert: 1 piece of fruit (200g) or flavoured gelatin (1/2=40g)
Snack:
One piece of fruit (200g)
Fruit juice/ Infusion. A piece of fresh cheese. No other cheese
Dinner:
Salad: lettuce (100g), tomato (75g), corn (30g), Fresh cheese as much as desired. Oil and vinegar.
Infusion (pennyroyal, chamomile). Fruit juice.

It is necessary to take the recommended amount and remember it is very important:
1- **DO NOT** take any forbidden food. Arrive in a **FASTING STATE** the day of the study and with the desire to urinate.
2- **DO NOT** take any medication in the morning, **BRING IT** with you to take when the researchers decide.
3- If you have any doubts, please contact the research group referred to in your copy of the informed consent.
Thank you very much for your collaboration.
